# Feed efficiency in dairy sheep: An insight from the milk transcriptome

**DOI:** 10.3389/fvets.2023.1122953

**Published:** 2023-04-03

**Authors:** Aroa Suárez-Vega, Pilar Frutos, Beatriz Gutiérrez-Gil, Cristina Esteban-Blanco, Pablo G. Toral, Juan-José Arranz, Gonzalo Hervás

**Affiliations:** ^1^Departamento de Producción Animal, Facultad de Veterinaria, Universidad de León, León, Spain; ^2^Instituto de Ganadería de Montaña (CSIC-Universidad de León), Grulleros, León, Spain

**Keywords:** dairy sheep, feed efficiency, mammary gland, RNA-Seq, sPLS-DA

## Abstract

**Introduction:**

As higher feed efficiency in dairy ruminants means a higher capability to transform feed nutrients into milk and milk components, differences in feed efficiency are expected to be partly linked to changes in the physiology of the mammary glands. Therefore, this study aimed to determine the biological functions and key regulatory genes associated with feed efficiency in dairy sheep using the milk somatic cell transcriptome.

**Material and methods:**

RNA-Seq data from high (H-FE, *n* = 8) and low (L-FE, *n* = 8) feed efficiency ewes were compared through differential expression analysis (DEA) and sparse Partial Least Square-Discriminant analysis (sPLS-DA).

**Results:**

In the DEA, 79 genes were identified as differentially expressed between both conditions, while the sPLS-DA identified 261 predictive genes [variable importance in projection (VIP) > 2] that discriminated H-FE and L-FE sheep.

**Discussion:**

The DEA between sheep with divergent feed efficiency allowed the identification of genes associated with the immune system and stress in L-FE animals. In addition, the sPLS-DA approach revealed the importance of genes involved in cell division (e.g., *KIF4A* and *PRC1*) and cellular lipid metabolic process (e.g., *LPL, SCD, GPAM*, and *ACOX3*) for the H-FE sheep in the lactating mammary gland transcriptome. A set of discriminant genes, commonly identified by the two statistical approaches, was also detected, including some involved in cell proliferation (e.g., *SESN2, KIF20A*, or *TOP2A*) or encoding heat-shock proteins (*HSPB1*). These results provide novel insights into the biological basis of feed efficiency in dairy sheep, highlighting the informative potential of the mammary gland transcriptome as a target tissue and revealing the usefulness of combining univariate and multivariate analysis approaches to elucidate the molecular mechanisms controlling complex traits.

## 1. Introduction

Feed costs represent a high proportion of total costs associated with the livestock industry [up to 65–70%; (1)]. Breeding more efficient animals would maximize farm profitability and also reduce the environmental impact of animal production (2). However, the challenges and costs of estimating feed efficiency make the implementation of this phenotype in animal breeding schemes difficult. Therefore, determining genes and biomarkers associated with feed efficiency could be of great interest to developing breeding strategies that can improve the feed efficiency of dairy cattle, leading to more sustainable and profitable dairy farming practices.

RNA sequencing is widely used in animal breeding to determine genes influencing complex traits, such as milk production, reproductive performance, and quality of carcasses (3). For feed efficiency, most studies using RNA-Seq data have been performed in pigs, chicken, and beef cattle [e.g. (4–6)]. In dairy sheep, hardly any transcriptomic studies have been conducted to characterize feed efficiency. To our knowledge, the only research aiming at identifying differentially expressed genes in sheep divergent for residual feed intake (RFI) values was published by Zhang et al. (7) using liver samples. A feed-efficient animal is generally defined as an animal that produces more while consuming the same amount of feed or that shows a lower intake for the same production level; therefore, as a simplified concept, feed efficiency is related to units of output product per unit of feed consumed (8). In this sense, when the final output of production is meat, it has been common practice for RNA-Seq studies to analyze muscle as the target tissue, both because of its importance for energy expenditure and because animals bred for meat production need greater muscle yield and higher conversion efficiency (9–13). In dairy ruminants, we have found only a few studies comparing divergent feed efficiency cows using liver and white blood cell transcriptomes: (14–17). However, we are not aware of any RNA-Seq approach linking the lactating mammary gland transcriptome and feed efficiency. Considering that the main productive goals of dairy animals are milk yield and milk fat and protein contents, previous studies have demonstrated the usefulness of using milk components to predict energy intake and efficiency in lactating dairy cows (18, 19). As higher feed efficiency means a higher capability of the animal to transform feed nutrients into milk and milk components, we hypothesize that high and low feed efficiency sheep would show transcriptomic differences in the mammary cells. In this regard, milk somatic cells have been demonstrated to be representative of the mammary gland tissue, which can be used as an effective approach to study the gene expression changes in the lactating mammary gland without performing biopsies (20). This is particularly important when looking for genes to be used as biomarkers of a complex trait, such as feed efficiency, mainly when the final aim of the research is knowledge transfer to commercial flocks.

Therefore, this study aims to characterize the transcriptome of the mammary gland in lactating sheep with divergent feed efficiency values by identifying genes, metabolic pathways, and biological processes potentially involved in this phenotype. At a practical level, concerning the interest in the dairy sheep industry, our objective is to obtain potential biomarkers for feed efficiency that can be used in the future in commercial population selection programs.

## 2. Materials and methods

### 2.1. Ethics statement

All experimental procedures were approved by the Research Ethics Committee of the *Instituto de Ganader*í*a de Montaña* (IGM), the Spanish National Research Council (CSIC), and the *Junta de Castilla y León* (Spain), following procedures described in Spanish and European Union legislation (R. D. 53/2013 and Council Directive 2010/63/EU).

### 2.2. Animals and sampling

This study constitutes a part of a larger research project aiming at providing new insights into the physiological mechanisms contributing to feed efficiency variation in dairy ruminants. A detailed description of the sheep management practices and samplings are detailed in Toral et al. (21). In brief, 40 Assaf ewes in the first half of lactation (mean body weight = 73.9 kg, SD = 8.9; days post-partum = 61.6, SD = 4.2; age = 3.4 years, SD = 1.4; milk yield = 2.6 kg/d, SD = 0.6) were housed in individual tie stalls, milked twice daily and fed ad libitum a total mixed ration (TMR) formulated from alfalfa hay (particle size > 4 cm) and concentrate (50:50). The TMR included molasses to hinder selection of dietary components. Results of chemical composition of the TMR have been reported previously (21).

Individual dry matter intake and milk yield were recorded over a 3-week period. Feed intake was calculated by weighing the amounts of dry matter offered and refused by each animal, and milk yield by weighing the total milk produced by each animal at morning and evening milking. Composite samples of the milk produced by each ewe were analyzed for fat, protein, and lactose concentrations. The body weight was recorded in two consecutive days per week.

For RNAseq analyses, we selected animals with divergent feed efficiency index (FEI), namely 8 of the least (L-FE group) and 8 of the most efficient (H-FE group) animals. Briefly, FEI was computed as the difference between the mean value of dry matter intake recorded (DMI_R_) and the mean value of predicted dry matter intake (DMI_P_) for the same period.


$$\begin{array}{l} FEI=DMI_{\text{R}}-DMI_{\text{P}} \end{array}$$

DMI_P_ was calculated as follows:


$$\begin{array}{l} DMI_{P}=ME_{mp}/ME_{TMR} \end{array}$$

Where ME_mp_ are the metabolizable energy requirements for maintenance, production, and body weight change (MJ/d), and ME_TMR_ is the metabolizable energy of the TMR (MJ/kg of DM). Both values were estimated using equations for metabolizable energy requirements for non-pregnant lactating sheep and TMR formulation and tables of the nutritional value of feed materials from the Agricultural and Food Research Council (22).

The higher the feed efficiency, the lower the value of the FEI, which averaged −0.29 (SD = 0.23) for H-FE, and 0.81 (SD = 0.24) for L-FE. As reported previously (21), this index showed a good agreement (*r* = 0.69, *P* < 0.01) with RFI in the same animals (estimated as the residual term from the regression of feed intake on various energy sinks: 0.16 ± 0.084 for L-FE, and −0.18 ± 0.082 for H-FE ewes).

Both groups of ewes, H-FE and L-FE, were sampled for RNA-Seq. Milk samples were obtained as described previously (23). Milk somatic cells (MSC) from healthy udders have been proven to be an alternative non-invasive approach for the lactating mammary gland transcriptome in ruminants with correlations of 0.98 with mammary gland biopsies (20, 24). To summarize, 50 mL of fresh milk was collected from each animal 1 h after milking and 10 min after injection of 5 IU of oxytocin/animal (Facilpart, Laboratorios SYVA, León, Spain) to maximize MSC concentration. To prevent RNA degradation, udders were cleaned with soap and water and disinfected with povidone-iodine, and the nipples were also flushed with RNAseZap (Ambion, Austin, TX, USA). A sterile gauze was used to cover the collection tube to avoid contamination.

For RNA extraction, MSC were pelleted by centrifugation at 650×*g* for 10 min at 4°C in the presence of a final concentration of 0.5 mM of EDTA. Then, the pellet was washed twice with 10, and 2 mL of PBS (pH 7.2 and 0.5 mM of EDTA) followed by centrifugation at 650×g for 10 min at 4°C.

The last pellet was kept in RNAlater (Sigma-Aldrich, Madrid, Spain) and stored at −80°C until RNA extraction using 500 μL of TRIzol according to the manufacturer's instructions (Invitrogen, Carlsbad, CA, USA). The RNA quality was evaluated using an Agilent 2,100 Bioanalyzer (Agilent Technologies, Santa Clara, CA, USA), obtaining a mean RNA integrity number of 8.2 (SD = 0.6; range = 6.8–9.1). The RNA sequencing was conducted at CNAG (Centro Nacional de Análisis Genómico, Barcelona, Spain), where the TrueSeq Stranded Total RNA Library Prep Kit (Illumina, San Diego, CA, USA) was used for library preparation. A HiSeq™ 3,000/4,000 sequencing system (Illumina) was used to generate stranded paired-end reads of 75 bp to a minimum depth of 30 million reads. Samples from six animals (4 L-FE and 2 H-FE), which did not reach the minimum depth required after the first round of RNA-Seq, were re-sequenced to achieve the minimum depth. Thus, there are two technical replicates for these six animals. The datasets generated for this study can be found in the ArrayExpress - EMBL-EBI database under the accession E-MTAB-12355.

### 2.3. Alignment and quantification

The alignment to the ovine reference genome (assembly ARS-UI_Ramb_v2.0) available at NCBI was performed using STAR v. 2.7.0 (25). For the alignment step, we used the following parameters: “–outFilterType BySJout” to reduce spurious junctions, “–outWigStrand Stranded” to indicate that our RNA-Seq data was stranded, and the option “– quantMode TranscriptomeSAM” to the necessary output for the quantification with RSEM v.1.3.3 software (26). The quantification of the gene expression for the different samples was carried out using RSEM v.1.3.3. The options applied for the quantification were “–paired-end” to indicate our data were paired-end, “–estimate-rspd” to estimate the start position of the distribution, “–calc-ci” to calculate 95% credibility intervals and posterior mean estimates, “–seed 12345” to set the seed for the random number of generators used in calculating posterior mean estimates and credibility intervals, and “–p 8” to fix the parallel environment.

### 2.4. Differential gene expression analysis

To perform the differential expression analysis (DEA), we first imported the samples into the R environment with the Tximport package (27). Once the matrix of counts per gene and sample was created, we kept genes with more than 10 counts in at least three samples (28). Then, technical replicates from the same sample were collapsed using the “collapseReplicates” function on DESeq2 (29). The DEA between L-FE and H-FE animals was performed using DESeq2 (29). We selected differentially expressed genes with a False Discovery Rate (FDR) <0.05, and log2FoldChange> |1.5|.

### 2.5. Sparse partial least square-discriminant analysis

The matrix of counts, normalized with DESeq2, was used to perform a sparse Partial Least Square-Discriminant analysis (sPLS-DA) in order to identify the key genes driving discrimination of our samples into H-FE and L-FE classes (30). To perform the sPLS-DA we used the R package mixOmics (30). We used the function “tune.splsda” to assess the optimal number of components and variables to select in each component. For this step, the function “tune” implements repeated (*N* = 10) and stratified (5-fold) the cross-validation to obtain the best predictive performance for the model. Then, the function “splsda” was used to classify the samples and select the variables. The function “vip” allowed us to obtain the variable importance in projection (VIP) coefficients, which reflect the relative importance of variables to explain each component. Genes with a VIP higher than two were selected for further analysis.

### 2.6. Functional enrichment analyses

Gene ontology (GO) terms and pathways were analyzed to explore the biological relevance of genes associated with feed efficiency in the DEA and sPLS-DA analyses. For the functional enrichment analyses, we used the ToppGene Suite (31), particularly the ToppFun functional enrichment tool. These analyses were performed for the different lists of genes obtained from the previous analyses, i.e., the differentially expressed genes (DEGs) and the list of genes obtained from the sPLS-DA analysis. The databases used for the enrichment were GO:Molecular function, GO:Biological Processes, GO:Cellular Component, and the Pathway databases included in ToppGene Suite (https://toppgene.cchmc.org/navigation/database.jsp), the full gene set of each database was used as background set for the analyses. The following options were applied to perform the functional enrichment analyses: “*P*-value Method = probability density function,” “multiple test correction = FDR,” “FDR cutoff <0.05,” “Gene Limits 2 ≤ *n* ≤ 2,000”. To visualize the results, the GOplot R package was used (32). First, we applied the GOplot function “reduce_overlap” to eliminate for each of the functional categories analyzed (GO:Molecular function, GO:Biological Processes, GO:Cellular Component, and pathways) the terms with a gene overlap ≥ 80%. Then, to plot the functional enrichment results, we used the “circle_data” GOplot function. This type of plot combines the results from the functional analyses with the log2 fold change of the genes within each GO term or pathway, and computes a *z*-score that indicates if the GO term or pathway is more likely to be upregulated (positive values) or downregulated (negative values) in H-FE ewes compared to L-FE ewes.

## 3. Results

### 3.1. Mapping statistics summary

An average of 43.24 (SD = 11. 98) million reads per sample (*n* = 16) was generated, the average million reads per library (*n* = 22; six animals had two technical replicates) was 31.4 (SD = 6.5 million reads). Overall, 96.65% of the reads aligned to the ovine genome; among them, 78.61% were uniquely mapped reads. A total of 15,116 genes were expressed (i.e., those detected in at least 3 samples and with 10 counts or more).

### 3.2. Differentially expressed genes between H-FE and L-FE sheep and functional enrichment analyses

In the DEA, 79 genes were identified as differentially expressed between H-FE and L-FE animals (FDR < 0.05 and log2FoldChange > |1.5|; Supplementary Table 1), 10 genes had higher expression in the lactating mammary gland of H-FE animals, and 69 had higher expression in the L-FE.).

**Figure 1:**
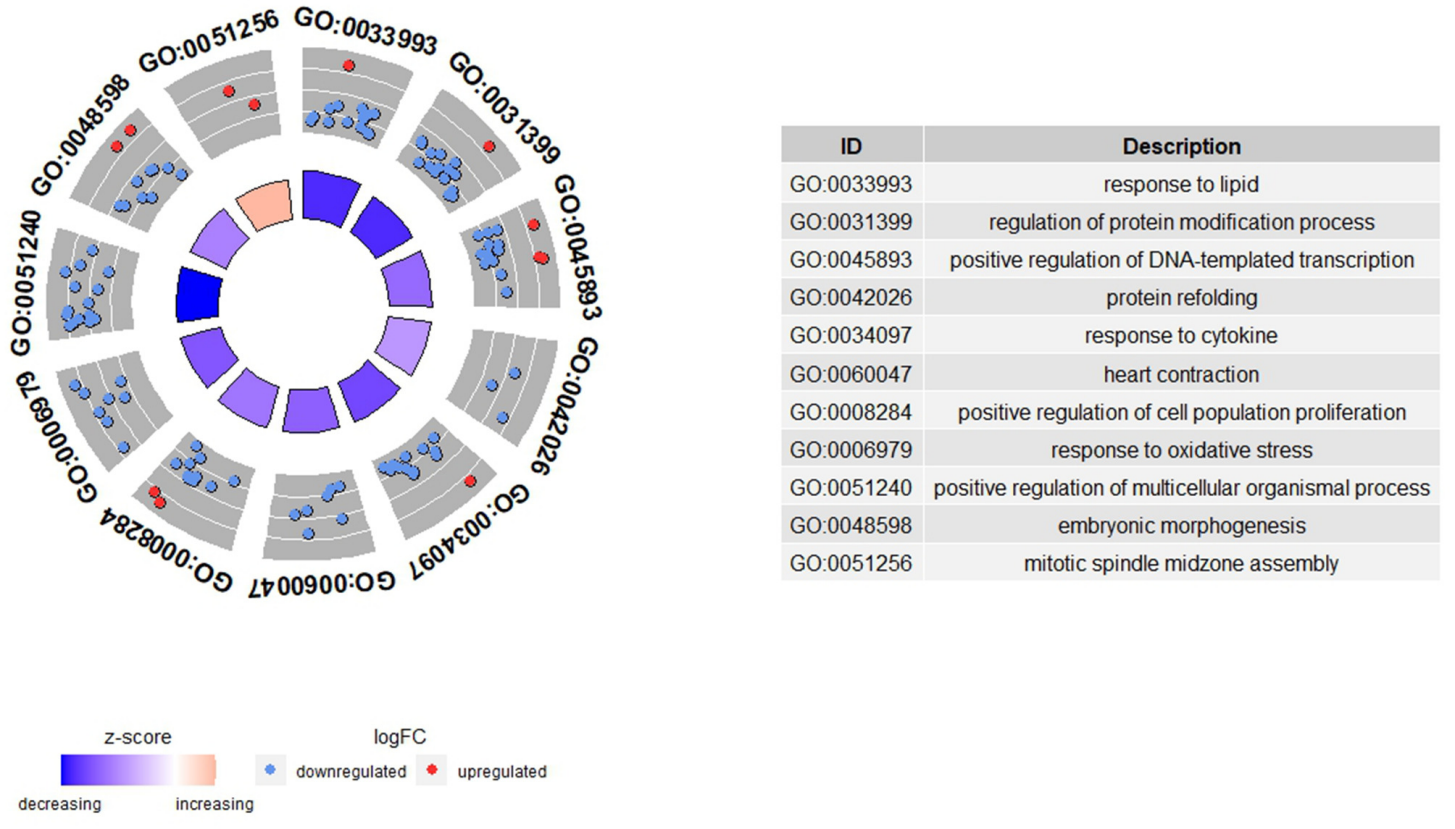
Functional enrichment results from the differential gene expression analysis between high (H-FE) and low (L-FE) feed efficiency animals. In the GOCircle plot, the significant GO terms enriched (FDR < 0.05) after a reduction of the terms with a gene overlap >80% are represented. The outer circle shows a scatter plot for each GO term of the logFC of the genes clustered in the term. The blue circles are genes downregulated in H-FE, while red circles are upregulated genes in H-FE sheep. The inner circle shows a bar plot representing the *z*-score for each GO term. The red bar means that the GO term is upregulated for H-FE, while the blue bar indicates the GO term is upregulated for L-FE.

Functional enrichment analysis was performed to determine which GO terms were enriched among the DEGs. We found enriched (FDR < 0.05) 27 GO terms in the biological process category (BP)-GO terms and two pathways [Source = MSigDB C2 BIOCARTA (v7.5.1)] (Supplementary Table 2). There was non-enrichment in the molecular function (MF) and cellular component (CC) categories. A total of 11 BP-GO terms remained after reducing the terms with a gene overlap >80% (Figure 1). The highest enriched BP-GO terms were “response to lipid” (16 genes; FDR = 1.637E-2), “regulation of protein modification process” (16 genes; FDR = 3.134E-2), and “positive regulation of DNA-templated transcription” (16 genes; FDR = 3.134E-2). All 11 BP-GO terms had a negative *z*-score, meaning they were downregulated in the H-FE animals, but the BP-GO term “mitotic spindle midzone assembly” had a positive *z*-score (1.41) and was enriched with two genes (KIF4A and PRC1) (FDR = 4.976E-2). The pathways found enriched were “Overview of proinflammatory and profibrotic mediators” (5 genes; FDR = 2.991E-2) and “p53 transcriptional gene network” (4 genes; FDR = 2.991E-2).

### 3.3. Discriminant genes between H-FE and L-FE

**Figure 2:**
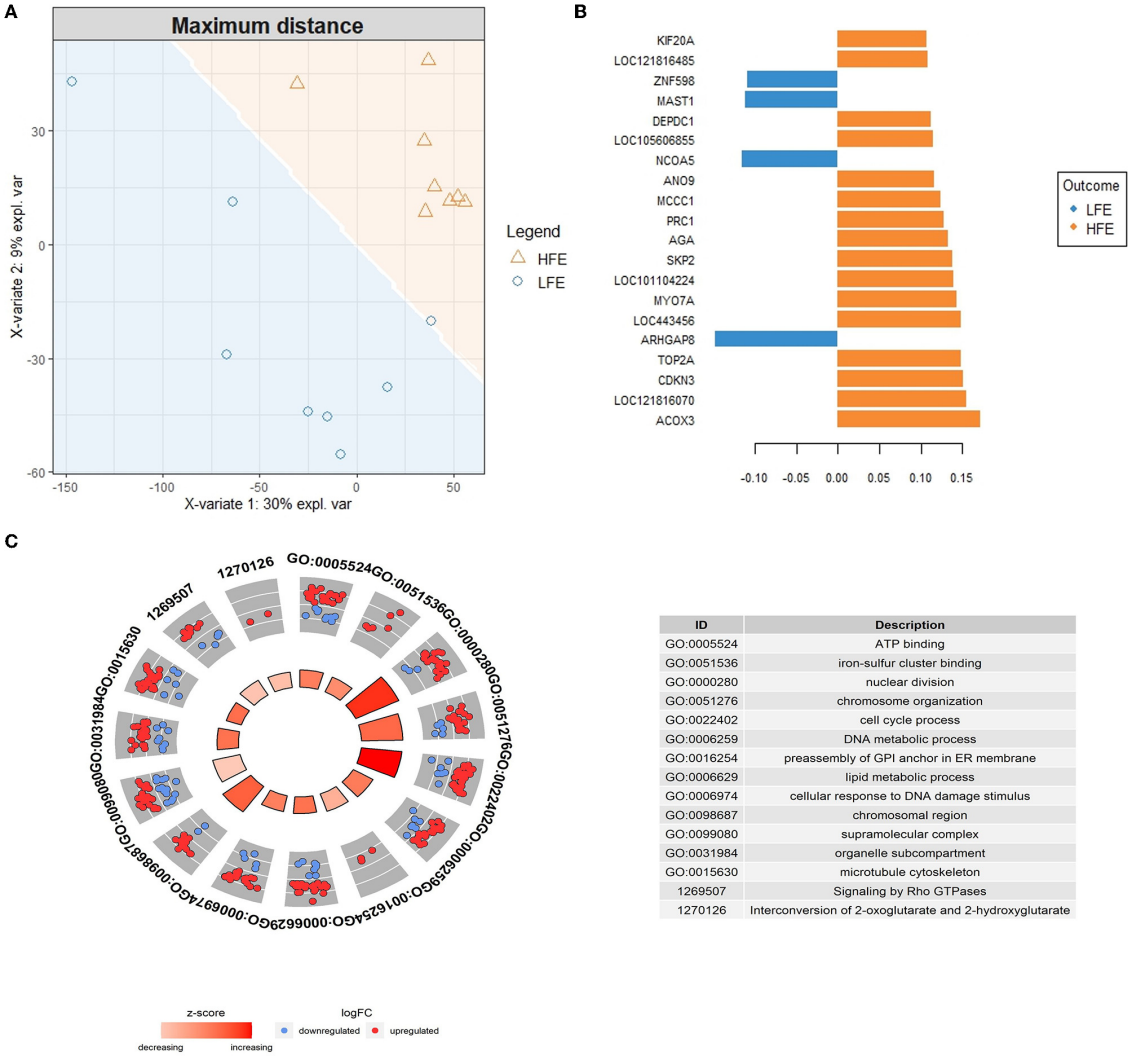
Results from the sparse Partial Least Square-Discriminant analysis (sPLS-DA). **(A)** Sample prediction area plot from the sPLS-DA model applied on the RNA-Seq data set from high (H-FE; orange triangles) and low (L-FE; blue circles) samples using as the distance for prediction “maximum distance”. **(B)** Loading plot of the top 20 discriminating genes on the first component between high and low feed efficiency animals, colors indicate the group in which the mean expression is maximal for each gene (H-FE: orange and L-FE: blue). **(C)** GOCircle plot showing the significant GO terms and pathways enriched (FDR < 0.05) after a reduction of the terms with a gene overlap >80% are represented. The outer circle shows a scatter plot for each term of the logFC of the genes clustered in the term. The blue circles are genes downregulated in H-FE, while the red circles are upregulated genes in H-FE sheep. The inner circle shows a bar plot representing the *z*-score for each term. The red color means that the GO term is upregulated for the H-FE group; the red color intensity is associated with the value of the *z*-score.

The supervised analysis with the sPLS-DA method was applied to discriminate between H-FE and L-FE animals (Figure 2A). The tune function led to an sPLS-DA model with one component and 380 predictive genes that could help to classify sheep as H-FE and L-FE; of them, 261 genes had a VIP > 2 (Supplementary Table 3). The prediction of the feed-efficiency status obtained with the first component was AUC (area under the curve) = 1 (*P*-value = 0.0007775), with a significantly balanced error rate of 0.32. The 20 genes with the highest loading are represented in Figure 2B. The loading weights were positive for the H-FE group and negative for the L-FE group. Interestingly, there were 15 genes overlapping between the sPLS-DA (VIP > 2) and the DEGs: *CCNA2, HS3ST1, HSPB1, IQCF1, KIF20A, KIF4A, LOC101111669, LOC101115355, MYO7A, NKX3*-1, *PDE4C, PRC1, PRDM5, SESN2*, and *TOP2A* (Table 1).

**Table 1:** Genes found in common by the differential gene expression analysis (DEA) and the sparse Partial Least Square-Discriminant analysis (sPLS-DA).

**Gene Name**	**DEA**			**sPLS-DA**	
	**FC**^a^ **(log2)**	***P*** **-value**	***P*** **-adj** ^b^	**VIP** ^c^	**Loadings** ^d^
*HS3ST1*	−3.22	4.89E-06	1.06E-02	2.32	−0.02
*PDE4C*	−2.70	2.47E-04	4.79E-02	7.63	−0.06
*IQCF1*	−2.51	2.05E-04	4.43E-02	5.27	−0.04
*HSPB1*	−2.20	8.32E-05	3.31E-02	2.78	−0.02
*NKX3-1*	−2.02	9.85E-05	3.70E-02	2.09	−0.02
*SESN2*	−1.67	1.12E-04	3.70E-02	7.08	−0.06
*LOC101111669*	−1.47	1.96E-04	4.28E-02	3.63	−0.03
*LOC101115355*	−1.06	1.18E-04	3.70E-02	11.83	−0.10
*PRC1*	1.19	1.93E-04	4.28E-02	15.71	0.13
*TOP2A*	1.49	1.85E-06	4.66E-03	18.29	0.15
*MYO7A*	1.67	2.90E-05	2.29E-02	17.56	0.14
*CCNA2*	1.75	7.10E-05	3.12E-02	10.10	0.08
*PRDM5*	1.76	1.64E-04	4.08E-02	5.93	0.05
*KIF20A*	1.85	1.43E-05	1.45E-02	13.10	0.11
*KIF4A*	1.91	1.30E-05	1.40E-02	13.04	0.11

a , fold change. Negative values correspond to higher expression in low feed-efficiency animals. Positive values correspond to higher expression in high feed-efficiency animals.

b *P*-adj, False Discovery Rate (FDR) multiple test correction performed by DESeq2.

c VIP, variable importance in projection.

d Loadings, value of the gene's loading weight (importance) on the first component of the sPLS-DA.

The functional enrichment analysis was performed with the discriminant genes between high and L-FE sheep with a VIP > 2. We identified 40 GO terms enriched in the BP category, five GO terms in the MF category, and 14 terms enriched in the CC category (Supplementary Table 4). The highest enriched terms in each GO category were “nuclear division” for the BP (26 genes, FDR = 1.154E-6), “ATP binding” for the MF (34 genes, FDR = 1.575E-2), and “chromosome, centromeric region” for the CC (15 genes, FDR = 7.342E-5). With the enrichment analysis using the pathway databases, 21 pathways were found to be significantly enriched (Supplementary Table 4), the highest enriched one “Cell Cycle, Mitotic” (22 genes, FDR = 1.454E-4, BioSystems: REACTOME). In Figure 2C, GO terms (2, 7, and 4 GO terms from the MF, BP, and CC categories) and pathways (2 pathways from the REACTOME database) remaining after eliminating those with a gene overlap >80% are represented. All non-redundant terms and pathways had a positive *z*-score, meaning that, in general, the genes clustered in each term/pathway had higher expression in the H-FE condition.

## 4. Discussion

The characterization of the genetic basis of economically relevant breeding traits is crucial to understanding the biology underlying these phenotypes and selecting animals with higher genetic merit. Regarding feed efficiency, RNA-Seq has been applied over the last decade to determine genes and markers related to this trait in several livestock species mainly intended for meat production [e.g., (6, 11, 23, 24)]. Nevertheless, less research has been performed in dairy cattle (14–16), and we are not aware of studies on dairy sheep. The liver has been the most commonly used organ to study gene expression differences in relation to feed efficiency (14–16). However, the collection of biopsies from an internal organ would not be feasible in practice. In this study, we analyzed RNA-Seq data from milk, which offers a novel perspective for the genetic characterization of feed efficiency. The milk transcriptome has successfully been used to examine differences in mammary metabolism due to breed (33), lactation stage (34), dietary lipid supplementation (35), and mastitis (36).

We used two bioinformatic approaches, DEA and sPLS-DA. DEA independently tests the expression level of each gene between conditions allowing the determination of the DEGs. Our study identified 79 DEGs between high- and L-FE sheep. Even though the number of DEGs is not high, a similar number of DEGs was identified in other studies using RNA-Seq technology to characterize differences in the transcriptome associated feed-efficiency, for instance when the liver was used as the target tissue [55 DEGs when studying FE for daily gain and body weight in Hu sheep (7) [FDR < 0.05 and log2FoldChange > |1.5|] and 70 and 19 DEGs (FDR < 0.05) for Holstein and Jersey dairy cattle breeds, respectively (16)]. These results give an idea about the complexity of feed efficiency, reinforcing the usefulness of using alternative tissues to gain biological insights about the trait further. Most of the genes found to be differentially expressed had a higher expression in the L-FE condition. Thus, most GO terms found enriched were related to the biological processes upregulated in the L-FE sheep. The highest enriched term among the DEGs was “response to lipid”, suggesting that dietary lipids can induce a different response in more or less efficient animals. Several studies in nutrigenomics in ruminants have demonstrated that lipid supplementation affects the lactating mammary gland transcriptome (35). Moreover, it has been shown that there is variability in the individual response to dietary unsaturated fatty acids (37). Although L-FE and H-FE ewes received the same TMR, a detailed fatty acid analysis of their ruminal digesta suggested a lower biohydrogenation extent of dietary fatty acids in the less efficient group (21), which might contribute to explain the present findings.

In addition, it is worth mentioning that almost all the genes in the GO term “lipid response” are also within the terms “response to cytokine” and “response to oxidative stress”. Metabolic adaptations to high energy demands, such as lactation, lead to lipid mobilization, which might be higher in less efficient sheep. This would be supported by the lower BW gain of the ewes over the trial and the lower milk *de novo* fatty acids/*cis*-9 18:1 ratio, a potential proxy of energy deficiency and body fat mobilization (21). Lipid mobilization has been demonstrated to favor inflammatory responses and oxidative stress (38). In addition, several RNA-Seq studies with different target tissues and species, such as beef (39), dairy cows (16), pigs (40), sheep (7), and poultry (41) support our results and highlight the association between feed efficiency and the immune system and stress, indicating that the latter processes may increase maintenance requirements and so reduce production in L-FE animals (42).

Regarding upregulated genes in H-FE sheep, we found the enriched term “mitotic spindle midzone assembly”, which is associated with anaphase and cell division (43). The vast majority of cell proliferation in the mammary gland occurs during its allometric growth before puberty and during pregnancy, with the number of secretory cells in the mature udder correlating with milk yield (44). It has been demonstrated in dairy cattle and mice that there is a constant but low proportion of cell division during lactation (45). The genes involved in “mitotic spindle midzone assembly”, *KIF4A* and *PRC1*, showed low abundance in our transcriptomic data [<10 fragments per kilobase per million mapped reads (FPKM)], which agrees with the results reported in cattle postulating that cell division during lactation is low. However, the higher expression of these two genes in H-FE animals suggests that cell division might also be higher than in L-FE, consistent with the observed differences in milk yield between groups (21).

Systems biology is particularly interesting when determining the genetic basis of complex phenotypes, such as feed efficiency. Co-expression network analysis has been successfully used to analyze the genetic architecture of feed efficiency by finding modules of highly co-expressed genes (15, 39, 40). sPLS-DA has been successfully applied lately for high-dimensional classification problems in genome biology. However, to date, no studies have exploited sPLS-DA to study transcriptomics behind feed efficiency. Over other multivariate methodologies, such as co-expression analysis approaches, one of the main interests of using sPLS-DA is that it aims to determine the most discriminant set of genes between the sample groups by applying discrimination analysis, variable selection and dimension reduction (30, 46) simultaneously. As a multivariate methodology, modeling transcripts as a set, sPLS-DA provides a more accurate picture of the context of the biological system and complements the findings obtained from univariate approaches. In addition, it has been shown to effectively discriminate between groups using OMICs data where the number of features far outnumbers the number of samples by selecting those features that clustered those groups, even when they are hidden among a large number of noise attributes (30, 46). The application of feature selection techniques not only facilitates the stratification and prediction of the samples, but also allows the identification of biomarkers related to the studied trait. In our analysis, the number of discriminant genes between H-FE and L-FE sheep was higher than the number of genes detected by the DEA approach (261 vs. 79 genes, respectively). These results allowed us to determine which biological processes were more relevant for the H-FE condition (Figure 2), complementing the results obtained by the DEA approach in which most enriched GO terms were associated with L-FE. The most enriched MF was “ATP binding”. Some studies in livestock species have related energy homeostasis production with feed efficiency (15, 40, 41). However, mutations in the ATP-binding domain have also been demonstrated to affect anaphase chromosome segregation in cultured cells (47). Thereunder, the majority of the terms enriched were linked to “nuclear division”, “chromosome organization”, and “cell division”, among others, with more than 20 genes clustered within these terms. This result highlights the importance of the findings previously discussed from the DEA analysis, suggesting a greater cell division in the lactating udder of H-FE sheep. Thus, we hypothesize that the higher milk yield of more efficient animals (21) could be due to a higher number of secretory cells. Moreover, the sPLS-DA methodology allowed the identification of a higher number of genes linked to cell division than DEA. This reinforces the use of a systems biology approach to understanding the complexity of the biological processes behind feed efficiency, which may be underestimated using univariate analyses (8).

Another GO term enriched and associated with H-FE was “cellular lipid metabolic process”, consistent with the greater milk fat yield in more efficient animals (21). Some well-known genes involved in mammary lipid metabolism were found as discriminant genes between H-FE and L-FE sheep: *LPL, SCD, GPAM*, and *ACOX3*. The *LPL* gene product, the lipoprotein lipase, is involved in the mammary uptake of plasma fatty acids (48) and, in cattle, an association between *LPL* abundance and maintenance of milk synthesis through lactation has been suggested (48). Regarding the stearoyl-CoA desaturase (*SCD*), an enzyme implicated in the desaturation of fatty acids, polymorphisms in the *SCD* gene have been associated with milk and protein yields in dairy cattle (49, 50). Another gene associated with lipid metabolisms was *GPAM*, which encodes for mitochondrial glycerol-3-phosphate acetyltransferase, a protein involved in triglyceride synthesis (51). In dairy cows, mutations in the *GPAM* gene were significantly correlated with changes in milk fat and protein or milk yield (52). The most discriminant gene between H-FE and L-FE sheep was *ACOX3*. This gene encodes for acyl-Coenzyme A Oxidase 3, which is involved in peroxisomal β-oxidation. The majority of cellular energy is supplied by the oxidation of carbohydrates, fats, or protein. Although *ACOX3* was upregulated in the H-FE group, in general, genes involved in fatty acid oxidation have been related to low feed efficiency in livestock (53) and negative energy balance (54), when using the liver as target tissue. However, oxidation in mammary tissue has received less attention, and its relationship with feed efficiency is thus less clear. In any event, genome-wide association studies in dairy cows suggested associations between *ACOX3* and fat percentage and some fatty acid concentrations in dairy cows (55, 56).

Finally, we conducted a literature review on those genes found in common by the two methodological approaches (DEA and sPLS-DA), or highlighted by studies on feed efficiency in other species. Some genes, such as *CRYAB, HSPB1*, or *PRC1* have been related to feed efficiency in livestock using other target tissues. The *CRYAB* gene, which encodes for Crystallin Alpha B protein, is upregulated in the liver, duodenum, and adipose tissue of L-FE pigs (40, 57), in the jejunum in cattle (58), and in breast muscle in poultry (59). This gene, and *HSPB1* (also known as *HSP27*), are members of the heat-shock protein family. The expression of heat-shock proteins increases as a cellular response mechanism to a stressor (60). *HSPB1* codifies heat-shock protein Family B (Small) Member 1, and contradictory findings have been found for this gene regarding feed efficiency. In beef cattle, higher expression of *HSPB1* has been associated with H-FE animals (61, 62), whereas in broilers, this gene is upregulated in L-FE animals (63). In our study, *CRYAB* and *HSPB1* were upregulated in L-FE sheep, which agrees with the results reported in pigs by Ramayo-Caldas et al. (40). Findings in dairy cows demonstrated that less efficient animals have higher heat production than efficient ones (64). Previous studies have revealed associations between SNPs in heat-shock proteins and traits such as respiration rate and body temperature (65, 66). Thus, it could be speculated that animals with a higher expression of heat-shock proteins are less efficient due to energy losses in greater heat production. Lastly, we would like to highlight the role of gene *SESN2* in lactation and its potential impact on feed efficiency, which could be a novel finding. *SESN2*, which codifies for sestrin2, belongs to a family of conserved, stress-inducible regulators of metabolism. A study on the influence of the expression of this gene on lactation suggested that *SESN2* negatively regulates cell proliferation and casein synthesis in cow mammary epithelial cells (67). Thus, the upregulation of *SESN2* in L-FE animals would lead to a decrease in milk yield. The fact that *SESN2* is involved in cell proliferation, such as other genes found in common by DEA and sPLS-DA approaches (specifically *KIF20A, KIF4A, TOP2A, NKX3*-1, *CCNA2, PRC1*), emphasizes the complementarity of the different methodologies applied and supports the potential relevance of mammary cell division for feed efficiency in dairy sheep.

## 5. Conclusions

The results from this study provide novel insights into the biological basis of feed efficiency in dairy sheep, highlighting the informative potential of the mammary gland as a target tissue and revealing the usefulness of combining univariate and multivariate analysis approaches to elucidate the molecular mechanisms controlling complex phenotypes. The DEA between sheep with divergent feed efficiency allowed the identification of genes associated with the immune system and stress in L-FE animals. In addition, the sPLS-DA approach revealed the importance of genes involved in cell division (e.g., *KIF4A* and *PRC1*) and cellular lipid metabolic process (e.g., *LPL, SCD, GPAM*, and *ACOX3*) for the H-FE sheep in the lactating mammary gland transcriptome. We also detected a set of genes commonly identified by the two statistical approaches, including some involved in cell proliferation (e.g., *SESN2, KIF20A*, or *TOP2A*) or encoding heat-shock proteins (*HSPB1*). Further research would be needed to elucidate the potential role of these genes as candidate biomarkers of feed efficiency in dairy sheep.

## Data availability statement

The data presented in the study are deposited in the ArrayExpress repository, accession number E-MTAB-12355.

## Ethics statement

All experimental procedures were approved by the Research Ethics Committee of the Instituto de Ganadería de Montaña, the Spanish National Research Council (CSIC), and the Junta de Castilla y León (Spain), following procedures described in Spanish and European Union legislation (R. D. 53/2013 and Council Directive 2010/63/EU).

## Author contributions

GH, PF, and PT designed the study with the cooperation of J-JA. GH, PF, and PT coordinated all tasks involved in animal maintenance, diet design, and phenotype recording. PT did RNA extractions. AS-V carried out the bioinformatic analyses. AS-V and BG-G wrote the first draft of the paper with the cooperation of PT, GH, PF, J-JA, and CE-B. All authors read and approved the content of the paper.
